# Self-regulated monitoring in undergraduate translation: a think-aloud study of SRL processes

**DOI:** 10.3389/fpsyg.2026.1887506

**Published:** 2026-09-10

**Authors:** Dan Xu

**Affiliations:** School of Advanced Translation and Interpretation, Dalian University of Foreign Languages, Dalian, China

**Keywords:** metacognitive monitoring, processing pressure, self-regulated learning, think-aloud protocols, translation process research

## Abstract

**Introduction:**

This exploratory qualitative study examines patterns of articulated monitoring depth in concurrent think-aloud protocols from undergraduate translation students, focusing on qualitative variation between surface monitoring (SM) and evaluative monitoring (EM).

**Methods:**

Twelve third-year undergraduate translation majors completed an English–Chinese literary translation task while verbalizing their thoughts concurrently. Participants were purposively selected from higher- and lower-scoring bands on a recent programme-wide translation assessment. Monitoring episodes were analyzed according to whether checking remained at local lexical or formal confirmation (SM) or involved testing candidate solutions against meaning, discourse relations, rhetorical purpose, or target-language fit (EM).

**Results:**

The central contrast concerned the regulatory endpoint of focal episodes rather than a uniform group difference. Several higher-scoring cases articulated sustained EM, whereas several lower-scoring cases included checks that ended once immediate lexical or structural uncertainty had been reduced. The distinction was neither categorical nor person-fixed: both forms occurred within the same participant, and P12 moved from dictionary-driven SM to discourse-based EM during the task. Within-case sequences further revealed narrowing, recovery, and maintenance of monitoring depth. Prolonged lexical search, repeated re-parsing, and unresolved uncertainty co-occurred with narrowing in some episodes.

**Discussion:**

Because cognitive load was not directly measured and concurrent verbalization may become less complete as task demands rise, these patterns are interpreted as hypothesis-generating rather than as evidence of a causal mechanism. Pedagogically, the findings support instruction that makes evaluation criteria, deliberate deferral, global rereading, and diagnosis-led revision explicit.

## 1 Introduction

Translation is a goal-directed problem-solving activity in which quality depends on how translators monitor, evaluate, and revise decisions while coordinating source-text comprehension, target-text formulation, and emerging discourse relations (Angelone, 2010; Jääskeläinen, 2000). Building on this view, Translation Process Research (TPR) has examined how translators regulate limited cognitive resources while making and updating choices during task execution (Alves and Hurtado Albir, 2010; Göpferich, 2009).

In the context of translator education, the pedagogical focus has traditionally remained on the acquisition of translation strategies (e.g., transposition, modulation, or paraphrasing). However, empirical evidence suggests a persistent strategy-performance gap: even when students are explicitly taught advanced translation strategies in modern instructional settings, many struggle to apply them effectively during actual tasks (Kiraly, 2014; Indarti, 2024). This discrepancy suggests that the mere possession of strategy knowledge is insufficient. One important source of variation may instead lie in monitoring quality—the moment-by-moment metacognitive process through which a translator tracks, evaluates, and adjusts cognitive actions (Orozco and Hurtado Albir, 2002; Pym, 2015).

Zimmerman's cyclical model of self-regulated learning (SRL) conceptualizes regulation as a recursive process unfolding across three phases—forethought, performance, and self-reflection—thereby offering a principled lens for examining how learners regulate cognition during complex tasks (Zimmerman, 2000; see also Panadero, 2017). Drawing on this model, the present study focuses on performance-phase self-monitoring and examines qualitative variation in monitoring depth during concurrent translation task performance.

The need to examine monitoring quality is further underscored by theories of limited cognitive capacity. Cognitive Load Theory emphasizes that working memory is limited and that complex performance depends on how task demands compete for this capacity (Sweller, 1988, 2011, 2023; Paas et al., 2003). Recent psycholinguistic evidence similarly shows that lexical interference and syntactic restructuring can intensify processing demands during translation (Kasap and Işik, 2025). In the present study, however, cognitive load was not measured directly. The theory is used only to interpret whether observable stretches of prolonged lexical search, repeated re-parsing, and unresolved uncertainty coincide with changes in the depth of verbalized monitoring.

Despite the proliferation of quantitative studies linking SRL scores to translation grades, qualitative evidence remains limited on how regulation unfolds during task performance. Most existing research relies on self-report scales, which may be affected by social desirability and may not reflect task-based behavior (Veenman, 2011). Concurrent Think-Aloud Protocols (TAPs) can make verbalizable aspects of ongoing problem solving, uncertainty, and revision available for analysis (Ericsson and Simon, 1993; Lörscher, 1991; Li, 2004). They do not provide exhaustive access to cognition, however, and demanding episodes may reduce the completeness of verbal reports. The present study therefore treats TAPs as evidence of articulated monitoring, not as a transparent record of all reasoning.

The present study therefore examines patterns of self-regulated monitoring in 12 undergraduate translation majors through a qualitative analysis of TAPs. Specifically, it seeks to answer:

RQ1: What qualitative contrasts in articulated monitoring are observed across cases drawn from higher- and lower-scoring programme-assessment bands?

RQ2: How do observable indicators of processing pressure co-occur with the narrowing, recovery, or maintenance of monitoring depth within cases?

By refining the coding of monitoring depth and examining within-case trajectories, this study contributes an episode-level account of how student translators regulate decisions during task performance. Cognitive-load theory remains a limited explanatory lens rather than a tested mechanism. The principal contribution lies in distinguishing local confirmation from criterion-based evaluation and in identifying process conditions that can be examined more directly in future multimethod research.

## 2 Literature review

### 2.1 SRL and monitoring in translation process research

Translation process research has increasingly shifted attention from end products to the processes and traces of decision making, revision, and problem solving as performance unfolds during task execution (Jääskeläinen, 2000; Göpferich, 2009). Within Zimmerman's social cognitive account, self-regulation is shaped by reciprocal interactions among personal, behavioral, and environmental factors (Zimmerman, 2000, 2002). This perspective is therefore well suited to analyzing monitoring as an activity that is contingent on the immediate task context and responsive to evolving demands, an emphasis that aligns with contemporary reviews describing SRL as enacted in context and dynamically realized during performance (Panadero, 2017). From a social-cognitive standpoint, monitoring can be specified more precisely as a feedback-driven regulatory process in which learner beliefs, task demands, and behavioral adjustments jointly guide ongoing choices (Zimmerman, 2000, 2002; Bandura, 1986). In translation, such agency is manifested in the translator's capacity to intentionally steer cognitive actions to resolve linguistic obstacles while maintaining communicative equivalence. Historically, the most influential models of translation competence, most notably those developed by the PACTE Group (2003, 2011) and Göpferich (2009), recognized the necessity of a strategic sub-competence or a psycho-physiological component. These models conceptualized the translator as a rational agent who employs strategies to compensate for deficiencies in other sub-competences. However, a significant theoretical limitation of these earlier models was their static nature. While they identified what constitutes translation competence, they offered limited insight into the dynamic mechanisms of how such competence is activated and sustained during a task. Zimmerman's (2000, 2017) Cyclical Model of SRL successfully addressed this gap by introducing a temporal dimension to cognitive control, delineating self-regulation into three recursive phases: forethought, performance, and self-reflection.

In the forethought phase, which precedes the actual drafting process, translators engage in task analysis and self-motivation beliefs. Task analysis involves goal setting and strategic planning, where the translator constructs a mental roadmap of the target text (Zimmerman and Schunk, 2011). In translation studies, this phase aligns with pre-translation analysis, where the translator assesses the purpose of the text and anticipates potential challenges. Empirical research has demonstrated that experts invest significantly more cognitive effort in this phase than novices, establishing a robust global plan that minimizes reactive errors during the later stages (Shreve, 2006).

The performance phase represents the heart of the translation activity, where self-control and self-observation have been increasingly emphasized. Self-control strategies, such as mental imagery, self-instruction, and attention focusing, allow the translator to maintain focus on the task amidst competing cognitive demands. Self-observation, primarily manifested through self-monitoring, serves as the cognitive feedback loop that detects discrepancies between the emerging translated text and the intended meaning (Hild, 2014; Indarti, 2024). This phase is particularly critical because it is in the performance phase that the translator's strategic intentions meet the reality of linguistic constraints. However, process-oriented research suggests that online monitoring may be reduced under high cognitive load, because translators must allocate limited attentional resources to both source-text comprehension and target-text production while the task unfolds (Chen, 2025; Jia et al., 2023; Rojo López et al., 2024; Wang et al., 2025). The final stage, the self-reflection phase, occurs after a translation unit or the entire task is completed. Translators engage in self-judgment comparing performance against goals and self-reaction adjusting feelings of self-efficacy. While often overlooked in pedagogical settings, this phase is vital for the long-term development of translation expertise. It is during self-reflection that translators learn how to learn, internalizing successful strategies and discarding ineffective ones (Zimmerman and Moylan, 2009; Chen and Wang, 2025; Wang and Zeng, 2026; Yang et al., 2025; Zheng, 2025).

Despite the theoretical appeal of Zimmerman's cyclical SRL model, its application in translation research has faced persistent methodological constraints. A substantial portion of existing work operationalises SRL through offline self-report questionnaires, typically drawing on established instruments in educational psychology such as the Motivated Strategies for Learning Questionnaire (MSLQ) (Pintrich and De Groot, 1990; Pintrich et al., 1991; Yang and Wang, 2020). While such measures can be useful for identifying broad associations between learners' reported regulatory tendencies and translation outcomes, they are limited when the aim is to explain how regulation unfolds moment by moment during translation. Translation performance is highly task-dependent and time-sensitive, with regulatory effort fluctuating in response to local problem density and uncertainty; consequently, retrospective surveys are unlikely to capture the online dynamics of monitoring and revision during task execution (Panadero, 2017; Veenman, 2011). In addition, self-reports are vulnerable to social desirability and recall biases, encouraging accounts of what learners believe they typically do rather than what they actually do under processing pressure (Veenman, 2011). These limitations motivate process-oriented methods—such as think-aloud protocols—to examine SRL as enacted regulation rather than as *post hoc* self-description.

### 2.2 Metacognitive monitoring

Metacognitive monitoring, often situated within the performance phase of Zimmerman's cycle, is widely regarded as a core mechanism of self-regulation during task performance. In the context of translation, monitoring is defined as the continuous process of comparing the evolving target text against the internal representation of the source text's meaning and the overarching translational goals (Orozco and Hurtado Albir, 2002; Pym, 2015). Effective monitoring allows the translator to detect translation problems—discrepancies that arise when linguistic or cultural equivalence is compromised—and triggers the application of compensatory strategies (Lörscher, 1991).

However, a key limitation in the literature concerns how monitoring is conceptualized and measured. Empirical work often operationalizes monitoring as a broad umbrella construct, which obscures meaningful qualitative differences in monitoring depth. Process-oriented research suggests that monitoring can vary across levels of experience and proficiency. Building on this limitation, the present study proposes a two-level account of monitoring: surface monitoring and evaluative monitoring. Surface monitoring is primarily reactive and localized; it focuses on low-level linguistic accuracy, such as spelling, basic grammar, and word-for-word correspondence. Although necessary, surface monitoring alone may be insufficient for discourse-level coherence or stylistic control (Angelone, 2010; Shreve, 2006).

In contrast, evaluative monitoring represents higher-order engagement. It involves what Kussmaul (1995) describes as critical detachment—the ability of the translator to step back and evaluate the target text from the perspective of the target audience and the source text's logic. Evaluative monitoring is characterized by questioning dictionary definitions, considering alternative renderings, and rejecting literal senses that conflict with context. This form of monitoring may be difficult for undergraduate students to sustain during demanding stretches of a task. The present study therefore asks how articulated monitoring varies as translation decisions unfold.

### 2.3 Cognitive resource constraints as an interpretive lens

The current study draws on Cognitive Load Theory (CLT) as an interpretive framework rather than treating cognitive load as a measured construct. CLT holds that working memory has limited capacity for processing new information (Sweller, 1988, 2011, 2023). Translation may require simultaneous lexical retrieval, syntactic parsing, discourse integration, and target-language formulation, but the presence and magnitude of load cannot be inferred from any single pause, lookup, or restart.

For undergraduate translators, literary texts may create substantial processing demands through lexical ambiguity, figurative relations, syntactic complexity, and target-language reformulation. Seeber's (2013) account suggests that low-level processing demands may compete with higher-order control. This proposition provides a possible explanation for changes in monitoring depth, but alternative explanations—including lexical knowledge, confidence, task familiarity, anxiety, and limits of verbal reporting—remain plausible in the present dataset.

Evaluative monitoring requires a translator to maintain a task representation, compare alternatives, and test them against meaning and discourse criteria. Surface confirmation may be easier to verbalize and sustain when local uncertainty is unresolved. The study therefore asks whether transcript sequences marked by prolonged search or repeated re-parsing also show a narrowing of articulated evaluation. Co-occurrence is treated as hypothesis-generating evidence, not as proof that resource depletion caused the observed monitoring pattern.

### 2.4 Synthesizing the research gap

Despite the separate advancements in SRL and CLT research, their integration within the field of translation studies remains remarkably scarce. Most SRL studies in translation pay limited attention to processing constraints, assuming that if a student is motivated, they will regulate effectively. Conversely, CLT research in translation often focuses on the product without examining the process of the regulatory behaviors.

There is therefore a need for process-oriented research that examines how monitoring depth varies alongside observable processing demands during translation. Using TAPs, the present study traces episode sequences in which monitoring narrows, recovers, or remains evaluative. It does not estimate cognitive load or test a causal effect. Instead, it identifies temporal patterns that are consistent with, but cannot demonstrate, a processing-pressure account and that can be tested in future studies combining TAPs with direct load measures.

## 3 Methodology

### 3.1 Participants and grouping

Twelve third-year undergraduate students majoring in Translation were recruited from the Faculty of Translation at a public university in mainland China. All were L1 Mandarin speakers, aged 19–21, enrolled in an advanced translation programme, and had completed more than two years of formal English-language and translation training. The sample comprised 11 women and one man. A separate standardized measure of English proficiency and a systematic inventory of extracurricular translation experience were not collected; the programme assessment described below is therefore used only to characterize relative prior translation performance.

Purposeful maximum-variation sampling was based on a routine programme-wide English–Chinese/Chinese–English translation assessment administered shortly before the TAP sessions. The assessment was scored from 0 to 100. The 12 selected cases were rank ordered, and the observed gap between scores of 89 and 88 was used to form two equal analytic bands: a higher-scoring band (HS: P1, P2, P4, P10, P11, P12; M = 91.00, SD = 1.79, range = 89–94) and a lower-scoring band (LS: P3, P5, P6, P7, P8, P9; M = 84.17, SD = 2.64, range = 81–88). This was an analytic split for contrastive case sampling, not a validated proficiency cutoff. The assessment was a routine programme measure; a formal reliability coefficient and independent validity evidence were not available for the present analysis. Accordingly, HS and LS refer only to relative programme-assessment bands and not to stable learner types or task-specific translation quality.

### 3.2 Instrument: think-aloud protocols (TAPs)

Concurrent think-aloud protocols (TAPs) were used to elicit verbalisable aspects of participants' online decision making during translation. Following Ericsson and Simon's (1993) procedures, participants completed a 20-minute practice task and were instructed to verbalize whatever came to mind without explaining it to the researcher. The researcher remained present but did not engage in dialogue; after extended silence, only a neutral reminder (“Please keep talking”) was used. Sessions were completed individually in one sitting and audio-recorded. No deadline or experimentally imposed time pressure was used.

### 3.3 Rationale for verbalizing in mandarin Chinese

Participants were invited to verbalize in Mandarin Chinese (L1) while completing the English–Chinese translation task. The primary rationale was to ensure that concurrent verbalization captured translation-related monitoring and decision making, rather than introducing an additional L2 speaking demand. From a cognitive-load perspective, requiring verbal reports in an L2 may increase extraneous processing demands and may alter the distribution of attention across decoding, planning, monitoring, and revision (Sweller, 1988, 2011, 2023). Allowing L1 verbalization was therefore intended to reduce additional reporting demands.

This choice is also consistent with earlier TAP-oriented discussions in translation studies. Methodological reflections caution that audible thought reporting may become less natural when participants must translate into or verbalize in a non-dominant language, potentially triggering code conflict and altering the process being observed (Toury, 2012). Experimental translation tasks have also commonly been conducted from a foreign language into the mother tongue (Jääskeläinen, 2000). Within this tradition, Mandarin verbalization was used to reduce the additional demands associated with reporting in an L2 and to facilitate expression of ongoing monitoring (Bernardini, 2001).

Finally, in the Chinese translation-process research context, L1 verbalization has been used to elicit richer process evidence for discourse-level monitoring, coherence testing, and stylistic deliberation (Li, 2004). The original Mandarin wording, including brief code-switches, was retained in the transcripts. Excerpts reported in English were translated by the researcher and checked line by line against the Mandarin transcripts to preserve the decision sequence and analytically relevant hesitation or repetition. No independent blind back-translation was conducted, a boundary acknowledged in the limitations.

### 3.4 The translation task and textual analysis

The study employed an English-to-Chinese translation task designed to elicit observable monitoring and revision during concurrent task performance. The source text was a 158-word literary excerpt from Sarah Orne Jewett's (1882) “Tom's Husband”, portraying a shift from idealized courtship to the practical realities of marriage. It contains polysemous lexical items and locally ambiguous structures that invite competing interpretations and discourse-level coherence checks. The term online in this study refers to the coordination of comprehension, formulation, monitoring, and verbalization while the task was underway; it does not refer to externally imposed time pressure.

All participants completed the paper-based task in a quiet classroom, using printed source texts and handwritten Chinese translations. Computers, online search, machine translation, and AI tools were unavailable; paper dictionaries were permitted. No strict time limit was imposed, and each session ended when the participant completed the translation in a single sitting. Session duration ranged from 8.4 to 41.5 min (M = 27.13, SD = 11.95), and Mandarin transcript length ranged from 753 to 3,732 Chinese characters (M = 2,423.83, SD = 1,051.51). These differences are reported because verbal output and opportunity to produce codable episodes were not uniform across participants.

### 3.5 Data transcription and coding framework

All think-aloud sessions were audio-recorded and transcribed verbatim in Mandarin Chinese. Transcription retained pauses, hesitations, repetitions, self-repairs, laughter, and code-switching. These features were treated as contextual information, not as direct measures of cognitive load. Handwritten drafts and final translations were digitized so that articulated evaluations could be compared with visible crossings-out, insertions, rewordings, and rereading-related revisions.

Analytically, the study adopted a hybrid deductive–inductive approach. Deductively, Zimmerman's cyclical SRL model served as a guiding framework for locating regulation across forethought (e.g., initial task orientation and goal setting), performance (online self-monitoring and control), and self-reflection (post-task evaluation) (Zimmerman, 2000, 2002, 2017; Panadero, 2017). Inductively, within the performance phase the analysis focused on monitoring as a situated, moment-to-moment regulatory process and developed a coding scheme that distinguishes monitoring by qualitative depth. Building on repeated patterns in the transcripts, monitoring during task performance was operationalised into two categories: surface monitoring (SM) and evaluative monitoring (EM).

Coding was conducted at the level of a monitoring episode, defined as a continuous verbal segment oriented to checking, evaluating, or revising a translation decision. An episode began when a participant signaled doubt, initiated rereading or dictionary consultation, proposed an alternative, or explicitly evaluated a solution; it ended when a solution was accepted, deferred, or the participant moved to another translation unit. Surface monitoring (SM) referred to articulated checking that remained local and form- or word-focused, such as dictionary-driven selection without explicit contextual testing or a local correction justified only by intuitive acceptability. Evaluative monitoring (EM) referred to articulated testing of candidate renderings against discourse meaning, logic, rhetorical relation, reader fit, or target-language appropriateness. Silence, a short utterance, a pause, or low transcript verbosity was not by itself coded as SM. Where the verbal record did not provide enough evidence to determine the regulatory criterion, the analysis did not infer the absence of evaluative reasoning.

Because cognitive load was not directly measured, prolonged lexical search, repeated local re-parsing, unresolved uncertainty, and clause restarts were treated only as observable indicators of processing pressure. Their temporal co-occurrence with narrowing, recovery, or maintenance of articulated monitoring was examined within cases. These indicators were neither combined into a load score nor used as proxies for a latent construct, and no causal mechanism was tested.

### 3.6 Trustworthiness and reliability

To enhance the trustworthiness of the analysis, coding was conducted iteratively with constant comparison across cases and monitoring episodes. Emerging interpretations were repeatedly checked against newly coded segments and against contrasting patterns across the higher- and lower-scoring programme-assessment bands, with particular attention to episodes that challenged preliminary assumptions or fell near category boundaries. A shared codebook specifying the criteria for surface monitoring (SM) and evaluative monitoring (EM) was used throughout.

Two coders trained in translation process research worked with a shared codebook. A stratified subset of three complete transcripts (25% of the 12 cases), including participants from both programme-assessment bands and different points in the range of transcript lengths, was independently coded. Agreement for the primary SM/EM code reached Cohen's κ =.85. Disagreements were resolved through discussion, and recurrent boundary cases were used to refine the decision rules before the remaining transcripts were coded.

Handwritten drafts and final products were inspected for visible revision traces that could corroborate an articulated evaluation. These traces were not used to infer unspoken cognition or to rate translation quality. The interpretation also recognizes a central TAP limitation: as task demands rise, participants may verbalize less completely, so an apparently surface-oriented episode may partly reflect reduced reportability rather than the absence of evaluative reasoning. The coding safeguards above reduce, but cannot eliminate, this confound.

Because session duration and transcript length varied substantially, raw SM/EM episode counts were not treated as directly comparable measures of monitoring prevalence across participants or groups. The analysis instead focused on the criterion, regulatory endpoint, and sequential development of episodes. Participant-level durations and transcript lengths are reported in Table 1, while the findings use structured coding displays and contrasting within-case trajectories to make the evidential basis transparent. Claims concern recurring qualitative patterns rather than statistical group differences.

**Table 1:** Participant profile and TAP session descriptives.

ID	Band	Sex/age	Programme score (0–100)	TAP duration (min)	Transcript length (Chinese chars)
P1	HS	F/20	94	19.0	2,199
P2	HS	F/20	89	16.3	1,467
P4	HS	F/19	91	41.2	3,698
P10	HS	M/19	90	41.5	3,732
P11	HS	F/20	92	31.9	2,868
P12	HS	F/20	90	26.1	3,713
P3	LS	F/20	85	36.7	2,254
P5	LS	F/20	86	36.9	3,313
P6	LS	F/20	82	15.2	1,367
P7	LS	F/21	88	37.9	2,427
P8	LS	F/20	83	8.4	753
P9	LS	F/20	81	14.4	1,295

HS and LS are relative programme-assessment bands. The six–six split followed the observed gap between scores of 89 and 88 and is not a validated cutoff. Durations and transcript lengths are reported to show variation in the opportunity to verbalize and produce codable episodes.

## 4 Findings and interpretive analysis

The findings are organized around the two research questions. The first concerns a recurring qualitative contrast in the regulatory endpoint of articulated monitoring, not a categorical separation between two types of learner. The second traces within-case sequences of narrowing, recovery, and maintenance around observable processing pressure. Cognitive load was not measured, and reduced verbal detail during demanding stretches remains a competing explanation.

All participants displayed at least some articulated monitoring, including dictionary consultation, syntactic reconsideration, rereading, and revision. The cross-case contrast concerned what these actions accomplished in the focal episodes. Several higher-scoring cases converted local uncertainty into explicit tests of meaning, rhetorical relation, or target-language fit; several lower-scoring cases ended a check once immediate lexical or structural uncertainty had been reduced. Both SM and EM occurred in both programme-assessment bands, and no participant was treated as a fixed monitoring type.

To enhance analytic transparency, Table 2 presents the excerpt-to-code chain for selected positive, negative, and boundary cases. The display is an audit trail of coding decisions rather than a frequency table.

**Table 2:** Coding pathway from think-aloud excerpts to themes across SRL phases.

SRL phase	Raw TAP excerpt (English translation from Mandarin TAPs)	Initial code	Subtheme	Theme
Forethought	P1: “This sentence is long, and the word order doesn't fit Chinese thinking. I'll translate the when-clause first, then adjust the rest.”	noticing syntactic mismatch; clause reordering plan	global task framing	Task Representation
Forethought	P4: “I'll read the whole text first… ‘other cases' should be made explicit in Chinese… and ‘one' doesn't need to be translated.”	global reading; explicitation; omission decision	rhetorical positioning of the ST	Task representation
Forethought	P8: “First I'll read through the whole text and circle words I'm not familiar with… the sentences are quite long with many commas; I'll probably need to split them—one English sentence may become two or three Chinese sentences.”	vocabulary marking; planning segmentation	survival-oriented planning	Lexical–syntactic constraints
Performance	P1: “Care can't be translated as ‘concern' here—it doesn't work. I checked the dictionary and it can also mean anxiety/burden; in this sentence it should be ‘the burdens of life doubled.”'	rejecting default meaning; justifying alternative by context	suppression of literal default meaning	Evaluative monitoring
Performance	P12: “Upon earth cannot just be ‘on the earth.' With heaven in the context, it means they didn't live a ‘heavenly' life—they were still living as ordinary people in the human world.”	figurative relation check; context-based inference	figurative coherence checking	Evaluative monitoring
Performance	P12: “What does affection mean? I don't even know this word… I have to look it up again… and keenly—let me confirm it… there are too many meanings.”	repeated lookup; meaning overload talk	uncertainty management by lookup	Surface monitoring
Self- reflection	P12: “They were still units in modern society… how should I revise this? Sigh—let me go back and reread from the beginning.”	triggered global reread; revision decision	revision gate/re-framing	Diagnostic reflection
Self- reflection	P1 (summary statement): “This sentence is very long… we can segment it at the semicolon.”	noticing processing pressure; choosing segmentation	managing processing demands	Diagnostic reflection

As Table 2 illustrates, the distinction concerns the articulated criterion and endpoint of an episode rather than the amount of activity or effort. Dictionary use, rereading, or revision could support either SM or EM depending on whether the participant tested a solution against a wider constraint.

### 4.1 Forethought phase

In response to RQ1, this subsection examines qualitative contrasts in how cases from the two programme-assessment bands constructed task representations in the forethought phase. Zimmerman's forethought phase concerns how learners interpret a task, set proximal goals, and select strategies before execution. In translation, forethought is not merely planning to translate; it is the formation of a workable task representation—what counts as a good solution, what risks must be managed, and where effort should be allocated. In the present data, forethought verbalizations were often brief, but some supplied explicit criteria that later guided monitoring and revision.

Throughout the excerpts, participants are referenced using anonymised identifiers in the format P followed by a number (e.g., P1). Participant 10 (P10) offers a comparably explicit forethought sequence that already embeds discourse-level intentions. Before drafting, P10 reports a full read-through and marks uncertain items and structural boundaries (e.g., highlighting going halves, distressing moments, upon earth, and obligations, and noting the semicolon as a segmentation cue). P10 then frames the text as “about marriage in modern life” and anticipates that the contrastive stance signaled by “But” should be retained in Chinese. This forethought is consequential because it treats the task as meaning construction under a rhetorical contrast, not merely lexical decoding, thereby establishing an evaluative criterion (“the contrastive intent should be translated out”) that can later guide monitoring and revision.

In several lower-scoring cases, forethought centered on vocabulary deficits and sentence length rather than on interpretive or rhetorical goals. P8 begins by circling unfamiliar words and describing sentence length and punctuation (“the sentences are quite long… may need segmentation… one English sentence may become two or three Chinese sentences”). This was a relevant strategy, yet the plan remained predominantly about managing anticipated difficulty rather than establishing criteria for evaluating meaning. During performance, the protocol became dominated by repeated decoding and local checking. This sequence is treated as a case-specific pattern, not as evidence that lower programme scores determine a particular form of forethought.

The forethought contrast also appeared in how participants positioned the source text rhetorically. P1, for example, quickly identified that the English sentence structure “is not like Chinese thinking,” and immediately proposed a syntactic re-ordering strategy (“put the when-clause first”). That is not yet monitoring, but it is an early commitment to meaning-driven restructuring rather than word-by-word substitution. P1 also anticipates pragmatic issues such as the referent of “friends,” noting that translating it as “two friends” would be misleading and suggesting “two people” instead. Such remarks indicate that the participant enters the task with an interpretive stance: meaning is constructed, not extracted. This stance becomes the foundation for evaluative monitoring later.

Taken together, the forethought evidence suggests differences in the task representations articulated by several participants. Some higher-scoring cases stated discourse-level goals that later served as monitoring criteria, whereas several lower-scoring cases foregrounded anticipated lexical or syntactic difficulty. This is a pattern within the present cases, not evidence that programme score determines task representation.

In the forethought phase, the most analytically useful distinction was whether planning supplied a criterion that could later constrain a translation decision. Global planning was not assumed to be inherently superior, and local planning could be effective when it supported later evaluation.

### 4.2 Performance phase

Zimmerman's performance phase includes self-control and self-observation. In translation, the most visible form of self-observation is monitoring: checking comprehension, testing candidate solutions, detecting mismatches, and deciding whether to revise or proceed (Angelone, 2010). The think-aloud data show that monitoring cannot be treated as a single behavior. Participants often checked, but what they checked, and how they justified the check, varied drastically. The present analysis therefore distinguishes two ends of a continuum: surface monitoring and evaluative monitoring.

This distinction echoes process research that links expertise to uncertainty management rather than to the frequency of tactics. In the present dataset, criterion-based evaluation appeared less sustainable in some episodes marked by prolonged decoding and unresolved local uncertainty. This co-occurrence motivates a processing-pressure hypothesis, but it cannot be separated from lexical knowledge, task familiarity, confidence, anxiety, or variation in verbal report.

#### 4.2.1 Evaluative monitoring in illustrative higher-scoring cases

Several higher-scoring cases contained evaluative episodes in which dictionary meanings were treated as hypotheses rather than accepted as ready-made answers. P1′s handling of “care” illustrates this pattern. The participant rejected the accessible sense of concern because it did not fit “the cares of life had been doubled” and selected a meaning closer to burdens after testing the alternatives against sentence meaning.

Similarly, P12′s reasoning about “upon earth” shows discourse-level evaluation. Rather than translating it literally as “on the earth,” the participant recognizes that the contrast with “heaven” calls for an idiomatic opposition (“heaven” vs. “the human world”), and proposes an interpretive rendering akin to “still living in the mundane world.” The reasoning is explicitly contrast-driven: the participant constructs meaning by mapping figurative structure, then checks whether the proposed Chinese phrasing captures that structure. This monitoring goes beyond idiom knowledge; it involves evaluating how clauses relate to each other during task performance.

The same participant provides another rich example of evaluative monitoring, particularly in how the participant moves from an intentionally crude first pass to a second pass oriented to rhetorical naturalness. Early in the session, P12 explicitly says the first pass is “like machine translation,” an admission that functions as a metacognitive label: the participant is aware that the current output is structurally deficient and requires later reworking. Later, during revision, P12 attempts multiple rephrasings to make the opening more natural (“However… like many things… ideals lose to reality”), rejecting earlier versions as “weird” or “too fake,” and searching for a Chinese formulation that preserves meaning while improving discourse flow. This iterative search is a hallmark of evaluative monitoring because it is guided by a criterion of naturalness and coherence that goes beyond lexical accuracy. The participant is not simply editing; the participant is evaluating the rhetorical adequacy of competing drafts and choosing among them.

Across these instances, evaluative monitoring is characterized by three recurring features. First, it is constraint-based: candidate translations are evaluated against context, contrast relations, and rhetorical intent. Second, it is iterative: the participant returns to earlier decisions, not merely to correct mistakes but to improve communicative fit. Third, it is suppressive: literal or default meanings are explicitly inhibited when they conflict with discourse logic. These features correspond closely to a performance-phase self-observation system that not only records what one is doing but judges whether it meets a goal—precisely the type of cyclical control that Zimmerman conceptualizes.

#### 4.2.2 Surface monitoring and boundary cases

Several lower-scoring cases also contained dense lexical search, grammar comments, and repeated checking, but focal episodes sometimes remained local and reactive. P8, for example, moved among literal renderings for the heaven–earth opposition and recognized that the relation was problematic, yet the articulated checking ended without a stable discourse-level reformulation. This is evidence of an unresolved regulatory endpoint, not proof that deeper reasoning was absent.

P12 is an important boundary case because the same higher-scoring participant displayed both SM and EM. Early dictionary-driven checks for “affection” and “keenly” remained local and did not culminate in contextual testing; later, a broader reread enabled P12 to reconstruct the figurative heaven–earth relation and justify an interpretive solution. This within-person movement shows that SM and EM are episode-level states shaped by the unfolding task, not fixed properties of HS and LS participants. It also cautions against reading the group labels as a dichotomy.

P8′s sequence illustrates why processing pressure is examined at the level of temporal co-occurrence. The participant initially recognized a discourse problem, but as alternatives multiplied and the clause was repeatedly re-parsed, the verbal record narrowed toward local sense selection. Because TAP completeness may also decline during demanding episodes, the sequence supports only a provisional interpretation of reduced articulated evaluation.

Surface monitoring should not be equated with low effort. Frequent checking can reflect substantial engagement while still ending without an explicit discourse criterion. Conversely, a short episode can be evaluative when the participant states a relevant standard and uses it to accept, reject, defer, or revise a solution. For this reason, raw frequency or verbosity was not used as a proxy for monitoring depth.

### 4.3 Self-reflection phase

Zimmerman emphasizes that self-reflection is not an endpoint; it shapes the next forethought cycle through satisfaction, causal attribution, and adaptive inference (Zimmerman, 2017). In the present data, self-reflection was most visible in revision talk such as rereading, rejecting an earlier choice, and reformulating. Its function varied across focal episodes. In some higher-scoring cases, reflection generated a new constraint for subsequent decisions (e.g., “this needs to align with the contrast”) and guided substantive revision. In some lower-scoring cases, reflection remained local or affective (e.g., “this is not smooth”) and did not articulate why the wording was problematic. These are qualitative contrasts in the available verbal evidence, not exhaustive measures of participants' unspoken reflection.

P10 illustrates reflection-as-control through contrast-driven re-framing. After the initial read-through, P10 explicitly evaluates what the sentence “should do” rhetorically—namely, to make the post-marriage shift from the ideal to the practical salient—and treats this as a translation constraint. Accordingly, P10 decides to restructure the clause and adds a contrastive rendering (e.g., making explicit that life “is not as ideal as imagined, but rather submits to reality”) to realize the intended turn. This is self-reflection feeding forward: the diagnosis (“the contrastive intent must be translated out”) generates an adaptive inference that directly guides revision choices beyond word-level smoothing.

P1's reflection is less explicit, but it serves a similar purpose: the participant periodically looks back to keep referents clear and the draft coherent as it develops. This back-checking helps ensure that earlier choices remain consistent with what the text is trying to say, rather than letting small, local decisions pull the translation off track. Even when consulting a dictionary, P1 is not simply searching for a word, but checking whether a candidate fits the meaning already established. This habit supports evaluative monitoring throughout the task.

In several lower-scoring focal cases, a closing read-through registered awkwardness but yielded little articulated diagnostic feedback for substantive revision. In P8′s closing rereading, for example, dissatisfaction is voiced in general terms (e.g., that a sentence is “not smooth” or “doesn't sound right”), yet the subsequent edits remain limited and unresolved lexical uncertainty persists. In such episodes, reflection flags a problem without articulating why the phrasing is awkward or which interpretive constraint should be revised. Reflection becomes more productive when it yields diagnostic feedback that can be translated into an adaptive inference (e.g., “my initial framing is misaligned with the contrast,” or “this expression needs a figurative rendering rather than a literal one”). When reflection remains primarily affective (“this feels odd”) without diagnosis, it may have limited capacity to reorganize subsequent monitoring and revision.

Revision-related evaluation also varied within and across participants. Some focal episodes generated diagnostic feedback that supported substantive revision, whereas others registered only general awkwardness. These patterns co-occurred with differences in local processing demand, but the protocols cannot determine whether the difference arose from cognitive load, knowledge, confidence, or the completeness of verbalization.

The evidence therefore supports monitoring depth as an episode-level analytic distinction. It does not establish a stable cognitive trait or a causal effect of cognitive load.

In the self-reflection phase, several higher-scoring focal cases generated diagnostic feedback that reorganized subsequent revision, whereas several lower-scoring focal cases registered awkwardness without articulating a diagnosis capable of guiding substantive change. The pattern was not uniform across participants.

With respect to RQ1, the recurring contrast was one of coordination and regulatory endpoint rather than categorical group membership. Several higher-scoring cases articulated reusable criteria across planning, monitoring, and revision; several lower-scoring cases resolved immediate uncertainty without articulating a wider test in the focal episodes analyzed. P12 and other boundary episodes show that both orientations can occur within one participant.

### 4.4 Within-case monitoring trajectories under processing pressure

RQ2 was examined through sequence evidence rather than by treating group differences as evidence of a mechanism. Transcript order was inspected before, during, and after stretches marked by prolonged lexical search, repeated re-parsing, restarts, or unresolved uncertainty. Three trajectories were analytically informative: narrowing from an evaluative concern toward local confirmation, recovery from SM to EM, and maintenance of EM through control moves.

P8 illustrates narrowing. The participant initially recognized that “earth” did not form a satisfactory counterpart to “heaven.” As local alternatives multiplied and the clause was repeatedly re-parsed, the articulated episode became increasingly occupied by lexical selection and ended tentatively without a stable reconstruction of the figurative relation.

P12 illustrates recovery. Earlier dictionary-driven checks remained local, but a later global reread restored a discourse-level criterion. The participant then rejected “on the earth” and reconstructed the expression as ordinary life in the human world. The sequence shows that surface checking did not permanently preclude evaluative monitoring.

P10 illustrates maintenance. A global read-through, early marking of uncertain items, segmentation at the semicolon, and provisional framing of the ideal-versus-practical contrast appeared to preserve a usable task representation. Later decisions could be tested against that same rhetorical criterion.

Table 3 summarizes these trajectories. The cases were selected to show different within-person developments, not to estimate their frequency in the sample.

**Table 3:** Illustrative within-case monitoring trajectories.

Case	Transcript sequence	Trajectory	Interpretive significance
P8	Discourse concern → multiplying lexical alternatives and repeated re-parsing → tentative local solution	Narrowing	Articulated evaluation became less sustained as local uncertainty remained unresolved.
P12	Dictionary-driven local checks → broader reread → reconstruction of the heaven–earth relation	Recovery	SM did not preclude a later return to criterion-based EM within the same participant.
P10	Global read and rhetorical framing → segmentation and provisional drafting → later decisions tested against the same criterion	Maintenance	Control moves appeared to preserve a usable task representation for continued EM.

The table summarizes contrasting sequences selected for process explanation. It does not estimate the prevalence of each trajectory.

Across the three sequences, processing pressure did not mechanically produce surface monitoring. The records are consistent with the hypothesis that unresolved local demands can make articulated evaluative monitoring harder to sustain, while segmentation, deliberate deferral, and global rereading may support recovery or maintenance. This remains an interpretive hypothesis because load was not measured, TAP verbalization may become less complete under demand, and alternative explanations were not experimentally controlled.

## 5 Discussion

The main contribution of the study is the episode-level distinction between local confirmation and criterion-based evaluation. The data do not show that higher-scoring students always monitor deeply or that lower-scoring students lack evaluative reasoning. They show a recurring contrast in the endpoint of articulated checks and, through cases such as P12, movement between SM and EM within the same translator.

Zimmerman's cyclical model helps organize these patterns across task orientation, online monitoring, and revision. The findings refine the performance phase by showing that monitoring can vary in depth and that an evaluative criterion may be established, lost, recovered, or reused as the task unfolds. The group comparison is therefore secondary to the process trajectory.

Processing pressure offers one possible account of why evaluation sometimes narrowed. Prolonged search and repeated re-parsing co-occurred with reduced articulated discourse testing in several sequences, a pattern compatible with psycholinguistic evidence that lexical interference and syntactic restructuring can intensify translation processing demands (Kasap and Işik, 2025). However, this pattern is not a direct measure of cognitive load. It may also reflect lexical knowledge, confidence, anxiety, task familiarity, or reduced capacity to verbalize complex reasoning while translating. The data therefore support a testable interpretive hypothesis, not a demonstrated causal mechanism.

P12 is theoretically informative precisely because the participant displayed both SM and EM. The case weakens any simple HS/LS dichotomy and supports a situated view of self-regulation: monitoring depth is reconstructed from one decision to the next. A participant may narrow under unresolved local uncertainty and later recover evaluation through rereading or a renewed discourse representation.

Taken together, the findings suggest that translation competence may include a regulatory dimension: the capacity to formulate a usable criterion, test alternatives against it, and return to it during revision. Whether processing pressure causally limits that capacity remains an open question for multimethod research.

## 6 Implications and limitations

### 6.1 Pedagogical implications

The episode-level contrasts observed in this study suggest that monitoring depth deserves greater pedagogical attention alongside the teaching of translation strategies. The data do not establish that monitoring depth causes higher translation performance, nor were the products generated during the TAP task independently scored. They do, however, show how local checking and criterion-based evaluation can lead to different regulatory endpoints during concurrent task performance. Pedagogically, this supports making evaluative criteria and revision rationales explicit.

A practical starting point may be to strengthen fluency in linguistic decoding before expecting sustained higher-order evaluation. Episodes dominated by syntactic parsing and lexical retrieval were sometimes accompanied by narrower articulated monitoring, but the study did not test whether increased automaticity would cause deeper monitoring. Training could nevertheless include structured practice in common bilingual shifts, clause restructuring, and frequent collocational patterns, followed by tasks that require learners to state and apply discourse-level evaluation criteria.

Given this possible processing constraint, instruction can also make the self-regulation cycle visible and usable across the task. Rather than relying only on product evaluation, process-oriented scaffolding can require a brief pre-translation plan, explicit monitoring checkpoints, deliberate deferral of unresolved items, and a revision log that records why a change was made. These practices train students to connect planning, online evaluation, and diagnosis-led revision without assuming that the present study has established a causal load mechanism.

Sustaining these practices also depends on how learners interpret breakdowns and recover from them. When students interpret difficulty as an unchangeable personal deficit, they may disengage from diagnostic revision. Instructors can instead frame errors in terms of adjustable process factors, such as an unstable task representation, missing coherence criteria, or premature commitment to a local solution. This pedagogical proposal extends beyond the present dataset and should be evaluated empirically.

### 6.2 Theoretical implications

Theoretically, this research extends the application of Zimmerman's (2000) social-cognitive model to translation by showing how monitoring depth can function as an integrative mechanism linking forethought, performance, and reflection during task execution. Rather than treating the phases as a simple sequence, the findings suggest that early task framing can shape the criteria used during monitoring, and that reflection can feed forward into subsequent revisions by stabilizing or revising those criteria. In this sense, monitoring depth provides a useful axis for describing how self-regulation is enacted moment by moment in translation tasks.

The study connects self-regulated learning with cognitive-load theory only at the level of a provisional explanatory hypothesis. It identifies transcript sequences in which local processing difficulty and reduced articulated evaluation coincide, but it neither measures load nor establishes resource depletion. The theoretical contribution is therefore the operational distinction between SM and EM and the description of narrowing, recovery, and maintenance trajectories.

### 6.3 Limitations and future research

Several limitations define the scope of the conclusions. First, the sample comprised 12 volunteers from one translation programme, and the HS/LS split was based on a single routine programme assessment. Individual scores and the analytic split are now reported, but a formal reliability coefficient and independent validity evidence for the assessment were unavailable. Separate standardized English-proficiency scores and systematic data on extracurricular translation experience were not collected. The group labels must therefore be read as relative sampling bands rather than psychometrically established proficiency categories.

Second, the study examined one English-to-Chinese literary narrative. Its polysemy, figurative contrast, syntax, and ironic movement may have made discourse-level evaluation particularly visible, whereas technical, legal, medical, audiovisual, or pragmatic texts may organize monitoring differently. Translation direction is also consequential. In the present L2-to-L1 task, source-text comprehension may have been especially demanding, while Chinese formulation drew on participants' L1 resources. In Chinese-to-English translation, lexical retrieval, syntactic encoding, idiomaticity, and target-language acceptability may place greater pressure on production. The SM/EM distinction may remain useful, but its distribution and triggers cannot be assumed to be the same across genres or translation directions.

Third, no deadline or experimentally controlled time pressure was imposed. The study concerns concurrent task demands within a single sitting, not performance under a time limit. Cognitive load was not measured through subjective ratings, dual-task methods, eye tracking, pupillometry, or other direct indicators, so the processing-pressure account remains hypothesis-generating and causal language is not warranted.

Finally, concurrent verbalization is both the source of evidence and a possible source of bias. Participants may verbalize less completely when a passage is demanding, and some evaluative reasoning may remain silent or become automated. Silence and low verbosity were not coded as SM, and drafts were used for corroboration, but the study cannot rule out under-reporting of evaluative thought. English excerpts were translated and checked against the Mandarin transcripts by the researcher without independent blind back-translation. Session duration and transcript length varied substantially, which is why raw episode counts were not compared as if they represented monitoring prevalence. Future studies should combine TAPs with keystroke logging, eye tracking, stimulated recall, direct load measures, independently validated performance assessments, and systematic background measures across genres and translation directions.

## 7 Conclusion

This exploratory study used concurrent TAPs to distinguish surface monitoring from evaluative monitoring in an English-to-Chinese literary translation task. Across the analyzed corpus, the main contrast concerned the endpoint of articulated checking: several higher-scoring cases tested local uncertainty against meaning, discourse, or target-language fit, while several lower-scoring cases ended focal checks after immediate uncertainty had been reduced. The contrast was not categorical, and both forms occurred within the same participant. P12, in particular, demonstrated movement from dictionary-driven SM to discourse-based EM.

Within-case sequences of narrowing, recovery, and maintenance suggest that monitoring depth changes as the task unfolds. Prolonged lexical search and repeated re-parsing sometimes co-occurred with narrowing, while segmentation, deferral, and global rereading accompanied maintenance or recovery. These observations generate a processing-pressure hypothesis; they do not demonstrate that cognitive load caused the change, and the limits of concurrent verbalization must remain part of the interpretation.

The study's most defensible contribution is therefore an operational, episode-level account of monitoring depth and a set of process trajectories for future testing. Translation pedagogy may benefit from making discourse criteria and diagnosis-led revision explicit, while future multimethod research is needed to establish when and why evaluative monitoring becomes more or less sustainable.

## Data Availability

The raw data supporting the conclusions of this article will be made available by the authors, without undue reservation.

## Appendix A

### Source Text Used 960 in the Think-Aloud Task

The following 158-word passage was reproduced exactly as administered to participants. Source: Sarah Orne Jewett, “Tom's Husband” (1882). But, as has been true in many other cases, when they were at last married, the most ideal of situations was found to have been changed to the most practical. Instead of having shared their original duties, and as school-boys would say, going halves, they discovered that the cares of life had been doubled. This led to some distressing moments for both our friends; they understood suddenly that instead of dwelling in heaven they were still upon earth, and had made themselves slaves to new laws and limitations. Instead of being freer and happier than ever before, they had assumed new responsibilities; they had established a new household, and must fulfill in some way or another obligations of it. They looked back with affection to their engagement; they had been longing to have each other to themselves, apart from the world, but it seemed that they never felt so keenly that they were still units in modern society.
